# Optimization of the pulse electric field assisted extraction of black rice grain for antioxidant and sirtuin1 enzyme stimulation activities

**DOI:** 10.1038/s41598-022-10272-2

**Published:** 2022-04-19

**Authors:** Nuttinee Salee, Wantida Chaiyana, Artit Yawootti, Srisuwan Naruenartwongsakul, Wannaporn Klangpetch, Ponjan Walter, Niramon Utama-ang

**Affiliations:** 1 Division of Product Development Technology, Faculty of Agro Industry, Chiang Mai University, Chiang Mai, Thailand; 2 Department of Pharmaceutical Science, Faculty of Pharmacy, Chiang Mai University, Chiang Mai, Thailand; 3 Department of Electrical Engineering, Faculty of Engineering, Rajamangala University of Technology Lanna, Chiang Mai, Thailand; 4 Division of Food Engineering Development Technology, Faculty of Agro-Industry, Chiang Mai University, Chiang Mai, Thailand; 5 Cluster of High Value Product from Thai Rice and Plant for Health, Chiang Mai University, Chiang Mai, Thailand; 6 Cluster of Innovation Food and Agro-Industry, Faculty of Agro-Industry, ChiangMai University, Chiang Mai, Thailand

**Keywords:** Plant sciences, Chemistry, Engineering

## Abstract

Cyanidin-3-glucoside (C3G) and peonidin-3-glucoside (P3G) in black rice grain (BRG) demonstrate many beneficial health effects, including antioxidant and anti-aging properties. This research aimed to study on pulsed electric field assisted water extraction (PEF-AWE) on BRG using pre-treatment technique, which was determined for enhanced yields of C3G and P3G, antioxidant and sirtuin1 enzyme stimulation activities. The effects of operating parameters for PEF-AWE (intensity of electric field, X_1_: 3–5 kV/cm, number of pulse, X_2_: 1000–3000 pulse and BRG/water ratio, X_3_: 0.1–0.5 g/mL) were determined using regression analysis and optimized PEF-AWE condition using the response surface methodology. Regression models showed the intensity of electric field and BRG/water ratio were the strong influence parameters significantly on C3G (*p* < 0.01). The results highlighted optimized conditions of PEF-AWE followed by 5 kV/cm, 3000 pulse and 0.5 g/mL leading to achieve higher C3G (92.59 ± 4.79 mg/g) and P3G (4.59 ± 0.27 mg/g) than no pre-treatment by PEF process, approximately 60%. Additionally, PEF extracts of BRG can modulate the ability of surtuin1 enzyme to deacetylate substrate proteins (26.78 ± 0.50 FIR). Thus, PEF-AWE can be applied to produce BRG extract as natural antioxidant compound and functional ingredient.

## Introduction

Several studies reported the composition and concentration of these natural antioxidant compounds of pigmented rice varieties^1^. The relation of pigmented rice pericarp on the potential health benefits are described in many researches, including strong antioxidant activities^2^, role of aging prevention^3^, significantly preventing memory impairment and reducing risk in Alzheimer's disease^4^. Recent studies have highlighted that polyphenols of pigmented rice have appeared as a coming source of natural anti-aging compounds. Pigmented rice could modulate life span-expansion via a mechanism of ROS reduction that relates to operation under the highly protected Sir2-dependent signaling pathways, with the human homologs NAD-dependent deacetylase sirtuin-1 (surtuin1)^5^. Moreover, the polyphenols have effect of anti-aging due to activation of via regulation of some signaling pathways, such as NF-κB^6^. Previously literatures represented that natural extract had able to activate sirtuin1 enzyme stimulation activities, such as polymethoxyflavonoids from Kaempferia parviflora^7^, drosophila melanogaster^8^, flavonoid glucoside from the fruits of lycium ruthenicun^9^, and roots of codonopsis pilosula^10^. Especially, anthocyanins consisted of cyanidin-3-glucoside (C3G) and peonidin-3-glucoside (P3G) in pigmented rice exhibited anti-inflammatory effects by inhibiting NF-κB and ERK/MAPK signaling pathway^11^. Therefore, black rice grains (BRG) are pigmented rice varieties which have high C3G and P3G that possibility lead to produce high potential material for natural antioxidant and anti-aging substance.

Anthocyanins in BRG are one of the water-soluble polyphenolic compounds. These natural pigments are defined by a C6–C3–C6 skeleton^12^ and plenty of abundant flavonoid glycosides^13^. Natural anthocyanins are less stable and disintegrated when reveal to high temperature, light, oxygen, enzymes and metal ions^14^. For these reasons, several extraction technologies have been determined for enhancing extraction of anthocyanins of BRG. The BRG was extracted by buffer solution (pH 3.2) under temperature 50 °C and 80 min, and were purified using membrane separation and resin adsorption^15^. The anthocyanin yield using ultrasound assisted extraction (UAE) was significantly higher than the conventional extraction process for purple black rice^16^. A combination of UAE and microwave-assisted extraction has been shown to increase phenolic compounds from black rice husk significantly^17^. Such extraction process may cause a loss of anthocyanin content and other phenolic compounds.

PEF treatment is one of the most encouraging novel physical methods to enchance the efficiency of extraction process, based on mass transfer phenomena with non-thermal technique^18^. PEF treatment was used to extract several compounds from various food materials, such as sugar beet juice^19^, cocoa bean shell and coffee silverskin^20^, moringa oleifera dry leaves^21^ and lingzhi (*Ganoderma lucidum*)^22^. Observed PEF treatment (2 kV/cm, 1000 pulses and 64 kJ/kg) was also investigated to extract antioxidant compounds from brown rice. It showed that the higher antioxidant activity of the PEF extract can be coresponsed to a higher cytotoxic activity against colorectal cancer cells than the untreated extract, because of the effect of PEF on the permeabilization of rice bran cells^23^.

Accordingly, the main purpose of this research was to illustrate that PEF-assisted extraction of black rice grain (BRG) allows enhanced yields of anthocyanins with relevant to antioxidant activities and investigate the possibility effect on activation of the NAD+-dependent protein deacetylase surtuin1. The parameters of the PEF-AWE consisting of electric field intensity (kV/cm), number of pulse (pulse) and BRG to water ratio (g/mL) were optimized by using response surface methodology (RSM) coupled with central composite design (CCD). The optimized condition was developed through RSM for the maximum of content of anthocyanin (C3G and P3G), total phenolic content (TPC), antioxidant activity and sirtuin1 enzyme stimulation activity.

## Materials and methods

### Materials and chemicals

BRG variety (*Oryza sativa* L.), local name Luem-Pua, was cultivated and harvested in 2018 by Research Center for Development of Local Lanna Rice and Rice Product, Chiang Mai University, Thailand, according to Thai Agricultural Standard Guideline on Good Agricultural Practice for rice^24^. The identification was done according to the Rice Department, Ministry of Agriculture and Cooperatives, Thailand (http://www.ricethailand.go.th). BRG was stored in ziplock bag and kept at − 20 °C prior to use. BRG was re-dried by hot air oven at 40 °C for 12 h and kept at 24.5 ± 2.1 °C until dehusked. 2,2′-diphenyl-1-picrylhydrazyl-hydrate (DPPH), 2,2′-azinobis 3-ethylbenzothiazoline-6-sulphonate (ABTS) and Folin–Ciocalteu reagent, 2,4,6 tripyridyl-s-triazine (TPTZ) were purchased from Sigma-Aldrich (St. Louis, MO, USA). Potassium persulfate (K_2_S_2_O_8_), ferrous sulfate (FeSO_4_), hydrochloric acid (HCl), sodium carbonate (Na_2_CO_3_), sodium acetate (CH_3_COONa) and acetic acid (CH_3_COOH) were purchased from RCI Labscan Co., Ltd. (Bangkok, Thailand). AR grade ethanol and HPLC grade acetonitrile were purchased from Merck (Darmstadt, Germany). C3G and P3G were purchased from S.M Chemical Supplies Co., Ltd. (Bangkok, Thailand). Surtuin1 activity Assay Kit (Fluorometric) ab156065 was purchased from Abcam, USA (in accordance with manufacturer’s instructions).

### PEF instrumentation

**Figure 1:**
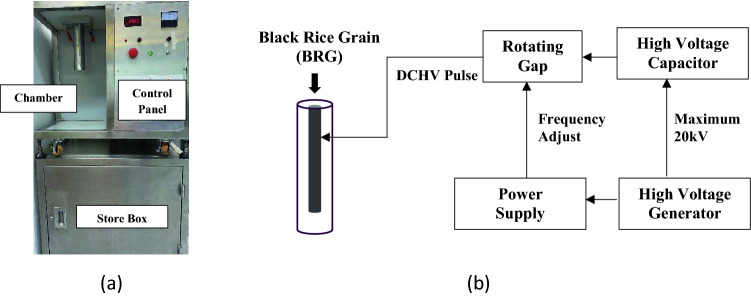
(**a**) PEF equipment and (**b**) schematic overview of PEF treatment system.

PEF treatments of various intensities and number of pulse were performed in a batch system; the PEF equipment and schematic overview are described in Fig. 1. Briefly, the electric field generator can generate maximum 20 kV that is operated by 220 VAC, 50 Hz and 500 W. Then, the energy is stored in a capacitor of 0.1 µF at a voltage of 20 kV before the rotating gap switches to supply a high voltage electric field (0–10 kV/cm and 1 µs) to the chamber. The extraction chamber is a coaxial-cylindrical PEF chamber with 20 mm and 60 mm inner and outer electrode diameter, respectively. Following the pulse electric field (PEF), the system can prefer the condition to electric field intensity of 2–6 kV/cm. with number of pulse from 100 to 4000 pulse.

### PEF-assisted water extraction (PEF-AWE)

**Figure 2:**
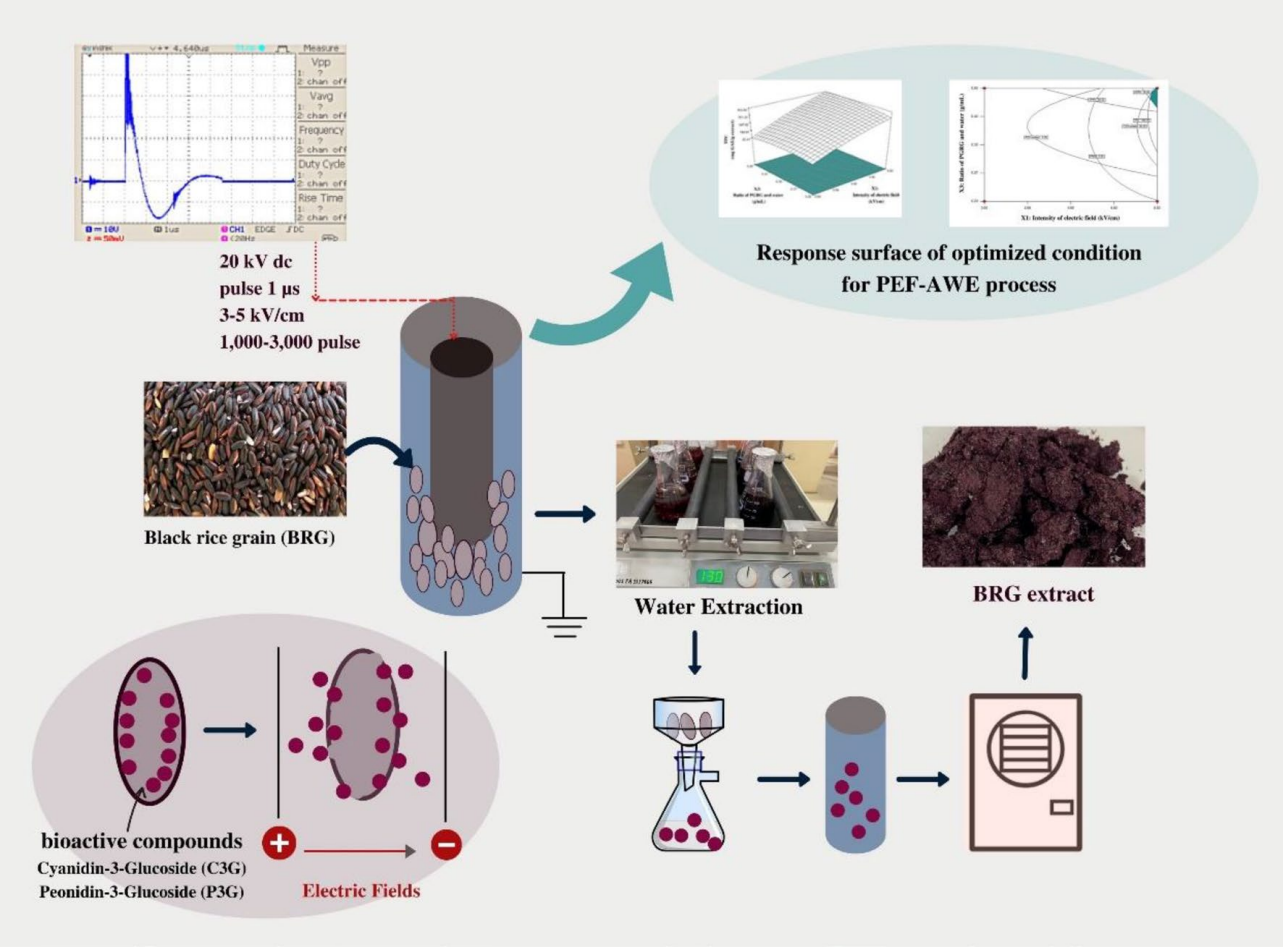
The process diagram of PEF-AWE.

After soaking in water for 10 min, BRG was loaded in the treatment chamber. The operating parameters of PEF-AWE followed by electric field was varied from 2.3 to 5.7 kV/cm, the number of pulses from 318 to 3681 at a constant pulse duration 1 μs and pulse repetition frequency 5 Hz. In all experiments, the initial temperature of the samples were 25.0 ± 1.3 °C. Then, the mixtures were extracted on an electrical shaker (Unimax2010, Heidolph) speed 150 rpm at 24 ± 2 °C for 6 h. The mixtures were filtered through filter paper (Whatman No.1, Merck, Germany) and freeze-dried, and then kept at 4 °C until use for analysis. The brief process diagram of PEF-AWE is shown in Fig. 2.

### Experimental design and statistical analysis

All Experiments were carried out according to the relevant guidelines and regulation. Experimental design and statistical analysis were performed by using Design-Expert software (version 6.0.10, Stat-Ease, Inc., Minneapolis, MN, USA). Three factor CCD consisted of 17 experiments (with three runs of center point) were applied to determine the optimal conditions. Intensity of electric field (X_1_), number of pulse (X_2_) and BRG/water ratio (X_3_) were independent variables. The code levels of the independent variables are given in Table 1. Mathematical models between variables were evaluated by means of multiple regression analysis^25^ in the following form:

1 $$ Y = \beta_{0} + \mathop \sum \limits_{i = 1}^{n} \beta_{i} X_{i} + \mathop \sum \limits_{i = 1}^{n} \beta_{ii} X_{i}^{2} + \mathop \sum \limits_{i = 1}^{n} \mathop \sum \limits_{j = i + 1}^{n - 1} \beta_{ij} X_{i} X_{j} + e $$

where $\beta_{0}$, $\beta_{i}$, $\beta_{ii}$, $\beta_{ij}$ are constant coefficients of regression, $X_{i}$ and $X_{j}$ are the independent variables, $Y$ is the dependent variable, $n$ is number of independent variables and $e$ is the random error term. The regression equations with coefficients and *R*^2^ values for each response were found by analysis of variance (ANOVA). For optimization, multi-response optimization methodology that maximizes the overall response functions was used.

**Table 1:** Code levels of independent variables for PEF-AE using CCD.

Independent variables	− *α* (− 1.68)	− 1	0	1	+ *α* (+ 1.68)
X_1_: Intensity of electric field (kV/cm)	2.3	3	4	5	5.7
X_2_: Number of pulse (pulse)	318	1000	2000	3000	3681
X_3_: BRG/water ratio (g/mL)	0.10	0.20	0.35	0.50	0.60

### Determination of C3G and P3G content

BRG extracts were separated and quantified with a high performance liquid chromatography (HPLC, Agilent Technologies, Santa Clara, CA, USA). A C18 rapid resolution column was used. The mobile phase was prepared with water, methanol and formic acid (75:18:7 v/v) with isocratic elution at 0.5 mL/min flow rate. The sample loop was 10 µL. The separated C3G and P3G were detected and measured at 510 nm^26^.

### Determination of TPC

TPC of BRG extract was determined by the Folin–Ciocalteu colorimetric method^27^. Briefly, 100 µL of 1:10 diluted Folin–Ciocalteu reagent was mixed with 20 µL of the extract solution (0.02 mg/mL) in a 96-well plate and incubated in the dark for 4 min. After that, 80 µL of 7.5% (w/v) Na_2_CO_3_ was added to the solution and incubated at 24 ± 2 °C for 2 h. The absorbance of the solution was measured at 760 nm using a microplate reader (DTX880, Beckman Coulter, Austria). Gallic acid standard curve was used (*R*^2^ = 0.997). The results of TPC were calculated as mg of gallic acid equivalent (GAE) per gram of extract.

### Determination of antioxidant activity

#### DPPH assay

The scavenging activity against DPPH radicals was determined according to a method described by^27^. Briefly, 180 µL of the DPPH solution was mixed with 20 µL of 0.001 mg/mL of extract solution and incubated in the dark for 30 min. The absorbance of the solution was measured at 520 nm. The scavenging activity was reported as % inhibition, which was calculated using the Eq. (2):

2 $$ \% {\text{ DPPH scavenging activity}} = \left( {\frac{C - S}{C}} \right) \times 100\% $$

where C was UV absorbance of control solution and S was UV absorbance of the sample solution.

#### ABTS assay

Scavenging activity against ABTS radicals was investigated following^27^. The mixture of 3 mL of 2.45 mM K_2_S_2_O_8_ and 2 mL of 7 mM ABTS solution were incubated in the dark for 24 h. Then 180 µL of the ABTS solution was added to 20 µL of the sample solution in a 96-well plate and incubated at room temperature for 5 min. The mixed solution was measured at 750 nm. The standard curve was constructed using Trolox (*R*^2^ = 0.993). ABTS scavenging activity was expressed as Trolox equivalent antioxidant capacity (TEAC), which represented the amount of Trolox (mol) equivalent per gram of extract.

#### Ferric reducing antioxidant power (FRAP) assay

Ferric reducing power was measured according to^27^. FRAP solution was prepared freshly by mixing 1 mL of 10 mM 2,4,6-TPTZ solution in 40 mM HCl with 1 mL of 20 mM ferric chloride and 10 mL of 0.3 M acetate buffer (pH3.6). For analysis, 20 µL of extract solution was mixed with 180 µL of FRAP solution. The mixed solution left to stand in a dark place for 5 min. The standard curve was constructed using FeSO_4_ (*R*^2^ = 0.999). Ferric reducing power was expressed as equivalent capacity (EC_1_), representing the amount (mM) of FeSO_4_ equivalent per gram of extract.

### Determination of anti-aging activity in term of surtuin1 enzyme-stimulating activity

Quantification of surtuin1 enzyme-stimulating activity was determined using the Abcam’s SURTUIN1 Activity Assay Kit (Fluorometric, ab156065) according to the manufacturer’s protocol. The solution of 25 µL of H_2_O (HPLC grade), 5 µL of surtuin1 assay buffer, 5 µL of fluoro-substrate peptide and 5 µL of NAD was added to microplate wells, respectively. Then 5 µL of sample was added into each well of the microplate wells. Initiate reactions were followed by adding 5 µL of developer to each well and mixing thoroughly at 24.0 ± 2.0 °C. Fluorescence intensity was read for 60 min at 2-min intervals using microplate fluorimeters (SpectraMax M3, Molecular Devices, USA) with excitation at 360 nm and emission at 485 nm. The stimulating activity of the extract on the surtuin1 enzyme was calculated according to^7^ as Eq. (3)

3 $$ {\text{Surtuin1 enzyme-stimulating activity}} = \left( {\frac{{FI_{sample} }}{{FI_{control} }}} \right) \times 100\% $$

where FI_sample_ and FI_control_ were fluorescent intensity of sample and control solution, respectively. Surtuin1 enzyme-stimulating activity was calculated in unit of ratio of fluorescent intensity (FIR).

### Scanning electron microscopy (SEM)

The physical surface structure of BRG was evaluated using a scanning electron microscope (Prisma E, thermo scientific, USA). BRG was placed on the surface of the stub. BRG was observed by SEM under an acceleration voltage of 10 kV, at a magnification of 3000× and 5000×.

### Fourier transform infrared (FT-IR) spectroscopy analysis

The FT-IR analysis was applied to identify the functional groups of the active components present in rice bran from the BRG before and after PEF-AWE. The FT-IR spectrum of samples in the wavenumber range of 4000–500 cm^−1^ with a resolution 4 cm^−1^ was obtained by FT-IR Spectrometer (FT/IR-4700, Jasco International Co. Ltd., Tokyo, Japan).

## Results

### Effect of PEF-AWE process variables on response values

C3G, P3G, TPC, DPPH, ABTS, FRAP to surtuin1 enzyme-stimulating activity, response values of BRG extract gained from PEF-AWE on CCD are listed in Table 2. The content of C3G and P3G of extract ranged from 21.825 ± 1.431 to 94.440 ± 1.408 and 1.026 ± 0.021 to 5.819 ± 0.053 mg/g extract, respectively. BRG extract through electric field intensity 4 kV/cm, number of pulse 3681 pulse and ratio 0.35 g/mL (run No. 5) has shown highest values for C3G content. TPC values for aqueous BRG extract varied from 16.096 ± 0.425 to 440.165 ± 7.135 mg GAE/g extract. The antioxidant activities were obtained by DPPH, ABTS and FRAP assay which their resulting values changed between DPPH (%inhibition) ranged from 18.542 ± 0.959 to 65.061 ± 0.808, ABTS (mol Trolox/g extract) ranged from 1.247 ± 0.375 to 3.700 ± 0.079 and FRAP (mmol FeSO_4_/g) ranged from 1.443 ± 0.077 to 5.657 ± 0.003, respectively. In addition, it was found that BRG extract clearly stimulated surtuin1 enzyme activity in unit of FIR varied from 2.37 ± 0.55 to 27.91 ± 0.36. It was observed that the combination of high intensity ranged from 4.5 to 5 kV/cm and BRG/water ratio ranged from 0.45 to 0.5 g/mL showed the high value of C3G and P3G content. It was found, three input variables affect the responses of extraction process; however, it is difficult to select the suitable condition or run of experiment in Table 2 to obtain high C3G, C3G, antioxidant and anti-aging activity from PEF-AWE process. Therefore, optimization using response surface modeling (RSM) was used for in this research.

**Table 2:** Experimental design and observed response of PEF-AWE process of BRG.

Run	Factor			Responses^b^						
	X_1_	X_2_	X_3_	C3G (mg/g)	P3G (mg/g)	TPC (mg GAE/g)	DPPH (% inhibition)	ABTS:TEAC (mol Trolox/g)	FRAP:EC_1_ (mmol FeSO_4_/g)	Sirtuin1 enzyme stimulation activities (FIR)
1	3 (− 1)	1000 (− 1)	0.5 (+ 1)	41.496 ± 0.299	2.821 ± 0.049	100.272 ± 1.064	35.012 ± 0.520	1.665 ± 0.046	1.543 ± 0.008	3.14 ± 0.36
2	3 (− 1)	1000 (− 1)	0.2 (− 1)	28.016 ± 0.861	1.026 ± 0.021	43.975 ± 2.182	30.059 ± 1.073	2.521 ± 0.009	1.443 ± 0.077	7.40 ± 1.05
3	3 (− 1)	3000 (+ 1)	0.5 (+ 1)	83.065 ± 1.401	4.417 ± 0.040	33.096 ± 0.831	52.304 ± 0.339	1.984 ± 0.602	3.533 ± 0.152	20.86 ± 0.34
4	3 (− 1)	3000 (+ 1)	0.2 (− 1)	73.541 ± 0.502	4.991 ± 0.024	42.281 ± 0.647	40.835 ± 0.623	1.263 ± 0.108	3.155 ± 0.083	10.29 ± 0.19
5	4 (0)	3681 (+ *α*)	0.35 (0)	94.440 ± 1.408	5.819 ± 0.053	289.762 ± 1.842	63.854 ± 0.825	3.030 ± 0.018	5.423 ± 0.084	24.43 ± 0.28
6	4 (0)	318 (− *α*)	0.35 (0)	21.825 ± 1.431	2.147 ± 0.071	79.236 ± 1.842	35.656 ± 0.488	1.820 ± 0.076	1.941 ± 0.234	7.27 ± 0.05
7	4 (0)	2000 (0)	0.60 (+ *α*)	45.173 ± 1.247	3.065 ± 0.195	224.270 ± 0.592	60.143 ± 0.648	2.227 ± 0.551	4.381 ± 0.120	8.28 ± 0.04
8	4 (0)	2000 (0)	0.10 (− *α*)	25.153 ± 3.062	2.123 ± 0.146	144.850 ± 2.127	38.081 ± 1.147	2.990 ± 0.045	3.501 ± 0.141	2.37 ± 0.55
9^a^	4 (0)	2000 (0)	0.35 (0)	60.972 ± 3.054	3.512 ± 0.058	258.446 ± 1.842	38.457 ± 0.924	2.360 ± 0.047	4.800 ± 0.141	5.33 ± 0.09
10^a^	4 (0)	2000 (0)	0.35 (0)	59.749 ± 2.397	4.075 ± 0.338	266.920 ± 5.848	38.414 ± 1.247	2.453 ± 0.261	4.855 ± 0.099	5.96 ± 0.43
11^a^	4 (0)	2000 (0)	0.35 (0)	60.508 ± 0.216	3.385 ± 0.152	283.007 ± 2.814	34.635 ± 2.730	2.479 ± 0.062	4.376 ± 0.148	6.03 ± 0.63
12	5 (+ 1)	1000 (− 1)	0.5 (+ 1)	46.596 ± 0.216	2.846 ± 0.098	214.929 ± 3.704	38.987 ± 1.262	2.531 ± 0.027	3.790 ± 0.119	10.26 ± 0.35
13	5 (+ 1)	1000 (− 1)	0.2 (− 1)	33.248 ± 0.484	2.291 ± 0.012	70.131 ± 1.842	42.993 ± 1.168	1.247 ± 0.375	3.480 ± 0.027	11.79 ± 0.48
14	5 (+ 1)	3000 (+ 1)	0.5 (+ 1)	90.431 ± 0.046	5.010 ± 0.192	440.165 ± 7.135	65.061 ± 0.808	3.700 ± 0.079	5.657 ± 0.003	27.91 ± 0.36
15	5 (+ 1)	3000 (+ 1)	0.2 (− 1)	76.578 ± 0.289	3.844 ± 0.039	357.305 ± 6.469	46.117 ± 1.645	2.958 ± 0.156	5.443 ± 0.007	15.79 ± 0.19
16	5.7 (+ *α*)	2000 (0)	0.35 (0)	94.206 ± 2.202	5.296 ± 0.089	380.638 ± 4.636	45.875 ± 2.566	3.911 ± 0.192	5.572 ± 0.194	24.41 ± 0.31
17	2.3 (− *α*)	2000 (0)	0.35 (0)	82.668 ± 1.798	3.604 ± 0.254	16.096 ± 0.425	18.542 ± 0.959	2.887 ± 0.146	1.911 ± 0.003	11.93 ± 0.60

X_1_ = Intensity of electric field (kV/cm), X_2_ = Number of pulse (pulse), X_3_ = Ratio of BRG and water (g/mL).

^a^Three runs of center point.

^b^Analytical results are the average of triplicates (mean ± SD).

### Model fitting and analysis of response surfaces

The ANOVA was performed to evaluate the predictive model (*p* < 0.05) and the variables as shown in Table 3. Additionally, the values of the determination coefficient (R^2^), adjusted R^2^ (Adj. R^2^) and predicted R^2^ (Pred. R^2^) were ranged between 0.87 to 0.99, 0.82 to 0.99 and 0.74 to 0.99, respectively.

**Table 3:** The regression equations based on ANOVA analysis of PEF-AWE process of BRG.

Model Variable	**C3G**		**P3G**		**TPC**		**DPPH**		**ABTS**		**FRAP**		Sirtuin1 enzyme stimulation activities (FIR)	
	Coefficient	*p* value	Coefficient	*p* value	Coefficient	*p* value	Coefficient	*p* value	Coefficient	*p* value	Coefficient	*p* value	Coefficient	*p* value
β_0_	89.46		− 2.85		− 437.62		15.43		2.47		− 11.45		83.85	
X_1_	73.73	< 0.0001	0.26	0.0102	149.36	< 0.0001	20.25	0.0002	0.35	0.0578	4.14	< 0.0001	− 33.22	< 0.0001
X_2_	0.03	< 0.0001	1.13 × 10^–3^	< 0.0001	− 0.055	< 0.0001	− 0.02	< 0.0001	0.29	0.1016	2.59 × 10^–3^	< 0.0001	− 0.02	< 0.0001
X_3_	328.77	< 0.0001	17.73	0.0446	892.20	0.0005	− 145.91	0.0006	0.044	0.7888	10.88	0.0083	− 48.62	< 0.0001
X_1_^2^	9.58	< 0.0001			− 29.91	0.0009	− 1.79	0.1323			− 0.38	< 0.0001	4.41	< 0.0001
X_2_^2^	− 1.13 × 10^–6^	0.0146			− 3.48 × 10^–5^	0.0004	4.41 × 10^–6^	0.0028			− 4.03 × 10^–7^	< 0.0001	3.59 × 10^–6^	< 0.0001
X_3_^2^	− 411.18	< 0.0001	− 22.16	0.0066	− 1546.18	0.0004	186.02	0.0038			− 13.82	0.0005		
X_1_ X_2_					0.073	< 0.0001								
X_1_ X_3_					150.45	0.0101							3.56	0.0327
X_2_ X_3_					− 0.11	0.0432	0.024	0.0185					0.02	< 0.0001
*p* value of the MODEL		< 0.0001^a^		< 0.0001^a^		< 0.0001^a^		< 0.0001^a^		0.0956^ ns^		< 0.0001^a^		< 0.0001^a^
Lack of fit		0.1715^ns^		0.3291^ns^		0.3003^ns^		0.2562^ns^		0.0087^a^		0.7961^ns^		0.2001^ns^
*R*^2^		0.9983		0.8650		0.9920		0.9496		0.6007		0.9873		0.9968
Adj. *R*^2^		0.9973		0.8200		0.9817		0.9105		0.3611		0.9797		0.9943
Pred. *R*^2^		0.9940		0.7427		0.9452		0.7878		0.7092		0.9528		0.9857

β_0_ = Intercept, X_1_ = Intensity of electric field (kV/cm), X_2_ = Number of pulse (pulse), X_3_ = Ration of BRG and water (g/mL).

^a^Significant (*p* < 0.05).

^ns^not significant (*p* > 0.05).

#### Effect of PEF-AWE process variables on C3G and P3G content

**Figure 3:**
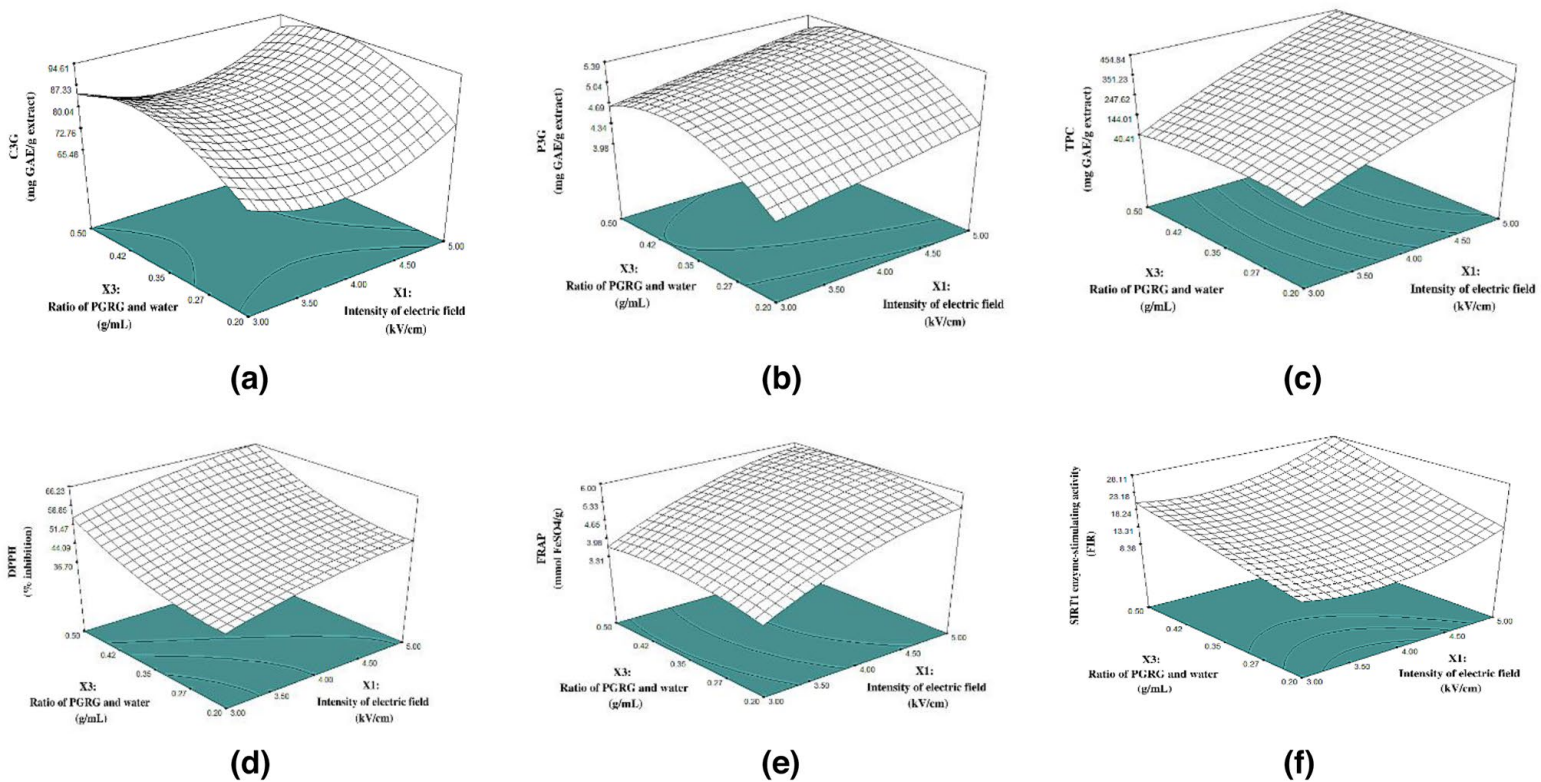
Contour plots effects between intensity of electric field (X_1_; kV/cm) and ration of BRG and water (X_2_; g/mL) with fixing number of pulse value at 3000 pulse on (**a**) C3G, (**b**) P3G, (**c**) TPC, (d) DPPH, (**e**) FRAP and (**f**) Surtuin1-stimulation activity respectively.

Table 3 represents that three factors affected significantly on C3G and P3G (*p* < 0.05). The content of C3G and P3G increased as all factors increased, which can be considered from their positive coefficients. And the regression coefficients from statistical analysis showed the intensity of electric field and BRG/water ratio were strong influence factors significantly on both of C3G and P3G of extract (*p* < 0.05). Response surface plot in Figs. 2b and 3a displays the interactive effect of electric field strength and BRG/water ratio on C3G and P3G, respectively.

#### Effect of PEF-AWE process variables on TPC and antioxidant activity

Modified quadratic model for different extraction conditions with R^2^ values (TPC = 0.9920; DPPH = 0.9496; ABTS = 0.6007; FRAP = 0.9873) are shown in Table 3. The small probability value of model (*p* < 0.0001) indicated that the TPC, DPPH and FRAP model were highly significant and could be used to forecast the response function accurately^28^. The large probability value of ABTS model (*p* > 0.05) was not significant. It was also observed that all of the independent variables affected the TPC, DPPH and FRAP of the extract significantly (*p* < 0.001). The *p*-value of regression equations indicated a strongly influenced interaction between intensity of electric field, number of pulse and ratio of BRG and water (X_1_X_2_, X_1_X_3_, X_2_X_3_) on TPC (*p* < 0.01).

#### Effect of PEF-AWE process variables on surtuin1 enzyme-stimulating activity

Table 3 shows the model obtained by RSM that presents relationships between extraction parameters and the surtuin1 enzyme-stimulating activity in terms of fluorescence ratio of intensity between extract and control. The correlation coefficient of R^2^ = 0.9968, Adj. R^2^ = 0.9943 and Pred. R^2^ = 0.9943 indicated acceptable correlation between the experimental values and model predicted by the equations.

### Optimization and validation of PEF-AWE process and water extraction

**Figure 4:**
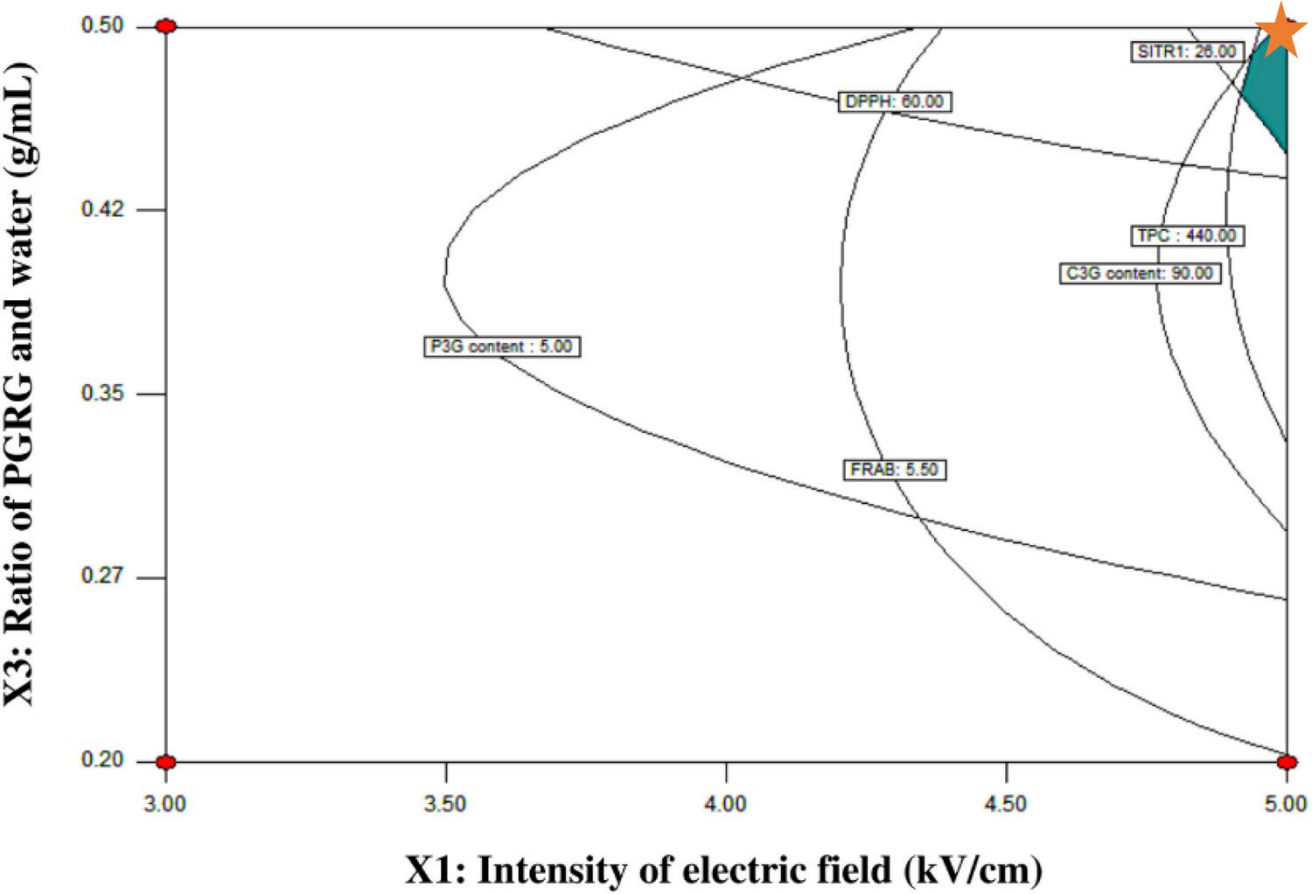
Response surface of optimized condition for PEF-AWE process with fixing number at pulse value of 3000 pulse.

Figure 4 shows the overlay plot of optimal PEF-AWE condition, which was generated using the high value of anthocyanin contents, antioxidant activity and surtuin1 enzyme-stimulating activity. PEF-AWE condition of electric field 5 kV and number of pulse 3000 at ratio of BRG and water 0.5 g/mL was observed. The values from the validation of PEF-AWE condition are shown in Table 4. Optimized pretreatment by pulse electric field of water extraction (PEF-AWE) and no pretreatment (water extraction) of BRG were compared after extraction as mentioned previously (Table 4).

**Table 4:** Validation of optimal condition of PEF-AWE process.

Response variables	Optimal condition^1^		Water extraction^2^	
	Predicted value	Observed values^3^	%Error^4^	Observed values^3^
C3G (mg/g extract)	90.27	92.59 ± 4.79^a^	2.57	57.43 ± 7.54^b^
P3G (mg/g extract)	5.17	4.59 ± 0.27^a^	− 11.16	2.80 ± 0.05^b^
TPC (mg GAE/g)	445.92	450.76 ± 10.14^a^	10.14	210.68 ± 18.02^b^
DPPH (% inhibition)	36.19	30.75 ± 2.06^a^	− 6.71	23.75 ± 0.73^b^
ABTS (mol Trolox/g)	Not include in predicted model	6.28 ± 1.67^ ns^	22.83	5.08 ± 0.96 ns
FRAP (mmol FeSO_4_/g)	5.84	7.89 ± 0.87^a^	35.15	7.67 ± 0.66^a^
Sirtuin1 enzyme stimulation activities (FIR)	27.97	26.78 ± 0.50^a^	− 4.27	10.09 ± 1.44^b^

^1^Calculated using the predicted equations for response variables. Given optimal condition of independent variables as intensity of electric field 5 kV, number of pulse 3000 pulse and ratio of BRG and water 0.5 g/mL.

^2^Distilled water, 6 h extraction time, 25 °C extraction temperature and ratio of BRG and water 0.5 g/mL.

^3^Means of three replicates experimental (mean ± SD).

^4^%Error = [(observed values − predicted value)/predicted value] × 100%.

^a,b^Values within the same row with different superscript letters are significantly difference and.

^ns^Indicated non-significant difference of observed values of optimal PEF-AWE condition and water extraction at *p* < 0.05.

### Effect of PEF on outer structure of rice

**Figure 5:**
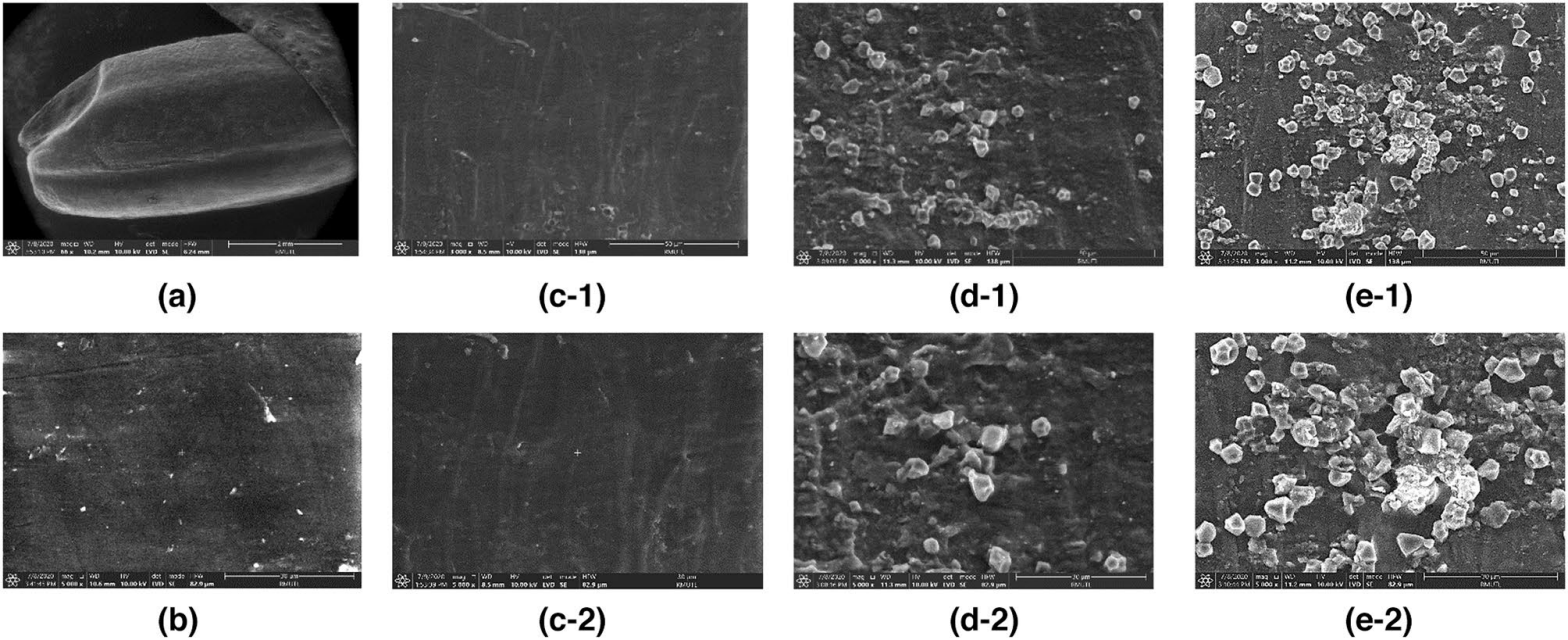
SEM of BRG during the PEF-AWE (**a**) position of *SEM analysis* (**b**) before soak in water in 5000x, (**c1-2**) after soak in water 30 min in 3000 × and 5000x, (**d1-2**) after pretreatment by PEF in 3000 × and 5000x, (**e1-2**) soaking process after PEF for 6 h in 3000 × and 5000x.

**Figure 6:**
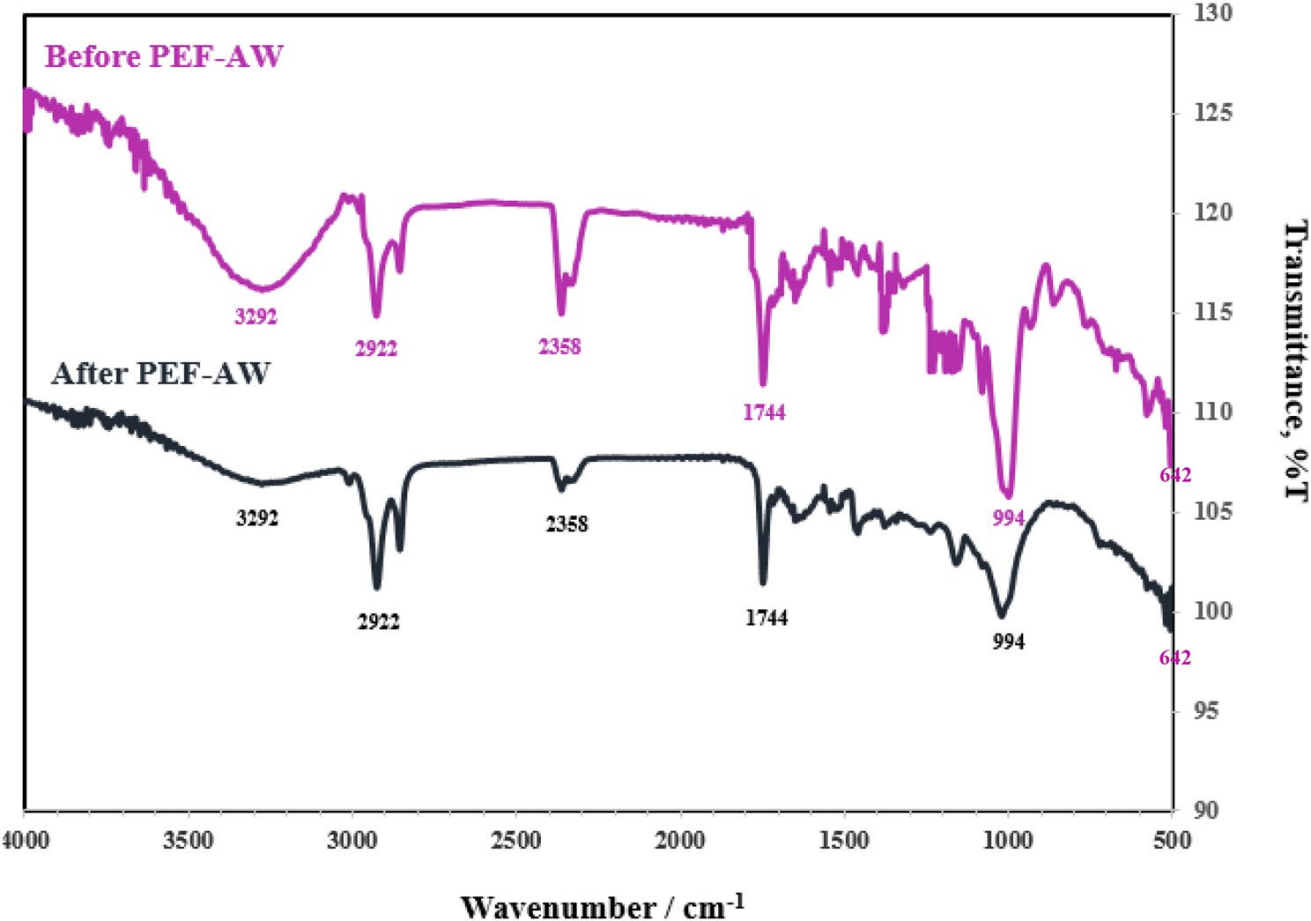
FTIR spectra of rice bran of BRG before and after extraction by PEF-AWE.

Figure 5a shows a detector position of SEM analysis on the outer surface of rice grain. The outer surface of non-treatment BRG appeared to be smooth and non-glistening, as shown in Fig. 5b. After soaking in water for 10 min, shown in Fig. 5c1–2, the grain surface becomes changed and not smooth, indicating that more starch swells during soaking^29^. Figure 6 shows FT-IR spectra of rice bran of BRG before and after extraction by PEF-AWE. The functional groups of the active components present in rice bran before and after PEF-AWE were identified by transmittance (%T) with purple and black line, respectively.

## Discussion

### Effect of PEF-AWE process variables on C3G and P3G content

This result shows a significant contribution of electric field strength (3.0 < X_1_ < 5.0 kV/cm) to increase the content of extractable bioactive compounds from BRG, due to the increasing of mass transfer through the cell wall of rice enclosing the bioactive compounds^23,30^. Our results were similar with study showing increasing the electrical field strength can increase the density of pores in the membrane and cell wall of onions^31^. This is confirmed by the changing of surface structure of the BRG during PEF-AWE in part of effect of PEF on outer structure of rice. However, while BRG was treated by PEF, temperature in the treatment chamber changed because of dissipation electrical energy. The final temperature of extraction was increased to 50.8 ± 1.7 °C. The temperature processing particularly above 50 °C or higher was possibly responsible for the result in degradation mechanism of anthocyanins^12^. For reduced thermal degradation of the C3G, P3G and others antioxidant compounds, BRG was drained in a bottle and suddenly cooled to 25 °C.

### Effect of PEF-AWE process variables on TPC and antioxidant activity

The regression coefficients from statistical analysis showed the intensity of electric field and BRG/water ratio were strong influence factors significantly on TPC, DPPH and FRAP (*p* < 0.05). The contour plots of interaction between intensity of electric field and BRG/water ratio are shown in Figs. 2e and 3c. In summary, the contour plots of two-factor interaction show that intensity 4.8–5.0 kV/cm, number of pulse 2800–3000 pulse and ratio of BRG and water 0.45–0.5 g/mL has significant influence to produce TPC value of extract more than 400 mg GAE/g. Additionally, interaction between number of pulse (X_2_) and ratio of BRG and water (X_3_) had significant effect (*p* < 0.05) on DPPH of extract, as shown in Fig. 3d. It showed increasing of number of pulse (X_2_ > 2000) and higher ratio of BRG and water (X_3_ > 0.42) which resulted in higher DPPH value. However, no significant effect was produced on PEF-AWE of DPPH and FRAP by the two-factor interactions (*p* > 0.05). Interestingly, this correlation was similarly reported of extraction process of bioactives from brown rice^23^. The research represented that the main factors of PEF treatment consisting of intensity and duration of pulse could contribute water-soluble antioxidants from brown rice grain, such as propionic acid, oxalic acid and isocitric acid, which have potential antioxidant properties.

### Effect of PEF-AWE process variables on surtuin1 enzyme-stimulating activity

It was observed that BRG/water ratio were the major critical factors (*p* < 0.01) for affecting the surtuin1 enzyme-stimulating activity. The regression equation did not present an interaction between intensity and number of pulse on the surtuin1 enzyme-stimulating activity (*p* > 0.05). Addition to interaction between intensity and ratio of BRG and water (X_1_X_2_) and interaction between number of pulse and ratio of BRG and water (X_2_X_3_) had significant effect (*p* < 0.05) on the surtuin1 enzyme-stimulating activity. The relationships between strongly two-factor interaction and surtuin1 enzyme-stimulating activity are presented in Fig. 3f. It is evident that the surtuin1 enzyme-stimulating activity increased with the increase in intensity of electric field and ratio of BRG and water at fixed number of pulse (3000 pulse). From the results, there is a noticeable point where the surtuin1 enzyme-stimulating activity has a corresponding change in the amount of C3G. This was related to the ability of C3G-induced peroxisome proliferator-activated receptor gamma coactivator 1-alpha (PGC-1a activity). This induction of PGC-1a gene expression was associated with increased surtuin1 gene expression^32^. Therefore, BRG extract has a probability to use as natural surtuin1 activators because flavonoid mixtures have been shown to induce surtuin1-mediated NF-κB inhibition^6^.

### Optimization and validation of PEF-AWE process and water extraction

The optimization of PEF-AWE condition (Run No.14 in Table 2.) provided the highest value of TPC (440.165 ± 7.135 mg GAE/g), DPPH (65.061 ± 0.808% inhibition) and FRAP (5.657 ± 0.003 mmol FeSO_4_/g), especially the greatest value of surtuin1 enzyme-stimulating activity (27.91 ± 0.36). Additionally, the PEF-AWE under optimized condition gained high content of C3G (90.431 ± 0.046 mg/g) and P3G (5.010 ± 0.192 mg/g) which approximate to results under Run No.5 (X_1_ = 4 kV/cm, X_2_ = 3681 pulse and X_3_ = 0.35 g/mL) and Run No.16 (X_1_ = 5.7 kV/cm, X_2_ = 2000 pulse and X_3_ = 0.35 g/mL). The results indicated that the moderate electric field strength between 4 and 5.7 kV/cm and number of pulse between 2000 and 3681 pulse with short pulses duration (t_PEF_ = 1 µs) abled to enhance anthocyanins content of BRG for pretreatment extraction process. This results were similar PEF-assisted extraction have higher extract yield of anthocyanins more than untreated samples^33^. However, the PEF pretreatment parameters in our research were optimized with type of raw material, targeted bioactive compounds and limitation of equipment.

In addition, the experimental under optimal conditions represented the close values of yield of C3G (92.59 ± 4.79 mg/g) and surtuin1 enzyme-stimulating activity (26.78 ± 0.50) with the predicted value less than 5% which shown in Table 4. With the optimal conditions shown antioxidant activities including DPPH (% inhibation), ABTS (mol Trolox/g) and FRAP (mmol FeSO_4_/g) value were 30.75 ± 2.06 and 6.28 ± 1.67 and 7.89 ± 0.8, respectively. The percentage of prediction error of C3G, DPPH and surtuin1 enzyme-stimulating activity were acceptable of 2.57, 6.71 and 4.27, respectively. Nevertheless, the percentage of prediction error of the other responses values were above 10%. Therefore the predictive performance of created model may be considered acceptable for C3G, DPPH and surtuin1 enzyme-stimulating activity because of the percentage of prediction error should not be above 10%^34^.

From the results obtained, the content of C3G and P3G of optimized PEF-AWE was higher than water extraction significantly by 61.22% and 63.92%, respectively. Because of higher anthocyanin content, it results in higher value of surtuin1 enzyme-stimulating activity. However, opposite trend appeared for the PEF-AWE and water extraction, as the ABTS and FRAP were not significantly different. This proves that pretreatment via PEF technique has a significantly higher C3G, P3G, antioxidant and surtuin1 enzyme-stimulating activity compared to water extraction.

### Effect of PEF on outer structure of rice

Water diffused into the rice kernel can improve the permeabilization index of rice grain and the electrical contact between the electrodes and the rice grain^23^. Then the surface of rice grain or pericarp was attached by PEF for 3000 pulse and 5 kV/cm (Fig. 5d1–2). Rough skin and holes were observed clearly on grain surface. Therefore, anthocyanin species and bioactive compounds that were packed and localized in the entire pericarp^35^ could be leached out effortlessly. Result of FT-IR analysis in Fig. 6 supported the enhancement of efficiency extraction of C3G and P3G by PEF-AWE. The vibrations of active components (C3G and P3G), which were the main components of rice bran, were different between before and after by PEF-AWE. Both of before and after PEF-AW displayed peak vibrations of the hydroxy groups at 3292 cm^−1^ (O–H stretching), which were the characteristic of phenolic ring^36,37^. Smaller peak intensities were noticed on the spectral data of the rice bran of BRG after extraction due to the lower concentration of hydroxy groups of phenols which rich in C3G and P3G. Moreover, the evidently decreasing of vibration band at 642 cm^−1^ (C–H bending), 994 cm^−1^ (C=C bending), 1744 cm^−1^ (C–H bending) 2922 cm^−1^ (C–H stretching) and were observed^38^. The results indicated the strength of pulse of electric field affect to unpacking structure of pericarp and increasing of C3G and P3G leached from pericarp in to water. Therefore, the surface of BRG can be destroyed by pulse electric field to obtain high C3G and P3G content. Additionally, the swelling compounds of starch granules were developed on the rice surface. The leaching out of compounds in pericarp and the swelling of starch granules continued as soaking process after PEF for 6 h. This is confirmed in Fig. 5e1–2 which shows more quantity and larger size of swell starch granules. Similarly, the losing of cell structure of rice grains and the starch granules was reported on pretreatment rice at 6 and 18 kV/cm by PEF^39^. According to the results reported in SEM and FT-IR analysis, PEF-AWE can improve extraction ability of BRG by attach grain surface and enhance diffusivity of interested components in pericarp of rice grain.

## Conclusion

In this study, PEF technique was used to enhance high biological extracts from BRG. It is shown that PEF-AWE which determined the optimum conditions of intensity of electric field 5 kV/cm, number of pulse 3000 pulse and 0.5 g/mL of BRG/water by RSM with CCD method proved effective in predicting the effect of three independent variables on content of C3G and P3G, antioxidant activity and anti-aging activity (surtuin1 enzyme-stimulating activity). Results indicated that the extraction yield of C3G, P3G and surtuin1 enzyme-stimulating activity from BRG was enhanced significantly by intensity of electric field. Addition to higher PEF strength combined with higher number of pulses at modulate temperature achieved higher antioxidant activity of the extract. Especially, content of C3G and P3G in PEF-AWE extracts was found of higher amount compared with no pretreatment by PEF extracts due to enhancing mass transfer through the pericarp of rice grain enclosing the bioactive components. It demonstrated that PEF-AWE has been shown as a potential technique to improve the extraction of valuable compounds from BRG.
